# Augmented Reality in Spine Surgery: A Case Study of Atlantoaxial Instrumentation in Os Odontoideum

**DOI:** 10.3390/medicina60060874

**Published:** 2024-05-27

**Authors:** Chi-Ruei Li, Yu-Jui Chang, Mao-Shih Lin, Hsi-Kai Tsou

**Affiliations:** 1 Department of Neurosurgery, Neurological Institute, Taichung Veterans General Hospital, Taichung 407, Taiwan; fantastic1694@gmail.com (C.-R.L.); mynewcellphoneyo@gmail.com (Y.-J.C.); ken70218@gmail.com (M.-S.L.); 2 Functional Neurosurgery Division, Neurological Institute, Taichung Veterans General Hospital, Taichung 407, Taiwan; 3 Department of Rehabilitation, Jen-Teh Junior College of Medicine, Nursing and Management, Miaoli 356, Taiwan; 4 Department of Post-Baccalaureate Medicine, College of Medicine, National Chung Hsing University, Taichung 402, Taiwan

**Keywords:** augmented reality, atlantoaxial instability, C1-C2 fixation, intraoperative CT, navigation surgery, os odontoideum

## Abstract

Despite advancement in surgical innovation, C1-C2 fixation remains challenging due to risks of screw malposition and vertebral artery (VA) injuries. Traditional image-based navigation, while useful, often demands that surgeons frequently shift their attention to external monitors, potentially causing distractions. In this article, we introduce a microscope-based augmented reality (AR) navigation system that projects both anatomical information and real-time navigation images directly onto the surgical field. In the present case report, we discuss a 37-year-old female who suffered from os odontoideum with C1-C2 subluxation. Employing AR-assisted navigation, the patient underwent the successful posterior instrumentation of C1-C2. The integrated AR system offers direct visualization, potentially minimizing surgical distractions. In our opinion, as AR technology advances, its adoption in surgical practices and education is anticipated to expand.

## 1. Introduction

Since Magerl and Seemann [1] proposed the technique of transarticular screw fixation for C1-C2 fixation, posterior instrumentation for atlantoaxial fixation has evolved significantly in the past few decades. In 1994, Goel and Laheri [2] presented a modified plate and screw method of fixation for 30 atlantoaxial dislocation cases. This technique was further modified by Harms and Melcher [3] with polyaxial screw and rod fixation. In recent decades, the C1 lateral mass to C2 pedicle screw-rod fixation technique (LC1–PC2 fixation) has gained widespread acceptance.

Despite the evolution and various adaptations of these techniques, the fixation of C1-C2 continues to pose a persistent challenge. Screw malposition and vertebral artery (VA) injury represent the most prevalent surgery-related complications in fixation procedures. To mitigate these life-threatening complications, various image-guided systems (IGSs) have been applied intraoperatively. These systems aim to enhance precision and safety during surgery. The use of intraoperative 3D image scanning, provided by cone beam-computed tomography (CBCT) or the O-arm system, has proven particularly effective. These advanced imaging technologies allow for the real-time visualization of the surgical field, enabling surgeons to avoid critical structures. By focusing on the prevention of VA, spinal cord, and nerve root injuries, these image-guided systems contribute significantly to improved surgical outcomes. The integration of such technologies into surgical practice underscores the importance of innovation in enhancing the safety and efficacy of C1-C2 fixation procedures, ultimately reducing the risk of complications [4,5,6].

However, traditional image-based navigation systems have primarily focused on establishing the spatial relationship between screw trajectory and bony structures, often leaving operators unable to visualize underlying vascular and neural structures. Another limitation of spinal navigation surgery lies in the need for attention shifting by the surgeon. During the navigation procedure, surgeons must frequently divert their gaze to external monitors displaying navigation images, which can lead to distractions and a loss of concentration.

To address these challenges, we introduce a microscope-based augmented reality (AR) navigation system that projects pre-set anatomical information and real-time navigation images directly onto the surgical field under the microscope. This innovative system integrates advanced imaging technology with the precision of a surgical microscope, enhancing the surgeon’s ability to navigate complex anatomical structures with greater accuracy and confidence. In this case report, we present a surgical case in which C1-C2 posterior instrumentation was successfully performed using this cutting-edge microscope-based AR navigation system. The system allowed for the precise placement of instrumentation while minimizing the risk of complications such as screw malposition and VA injury. By providing real-time feedback and visualization, the AR navigation system facilitated a safer and more efficient surgical procedure. This case underscores the potential of AR technology to revolutionize spinal surgery, offering a promising solution to the persistent challenges associated with C1-C2 fixation.

## 2. Case Description

**Figure 1:**
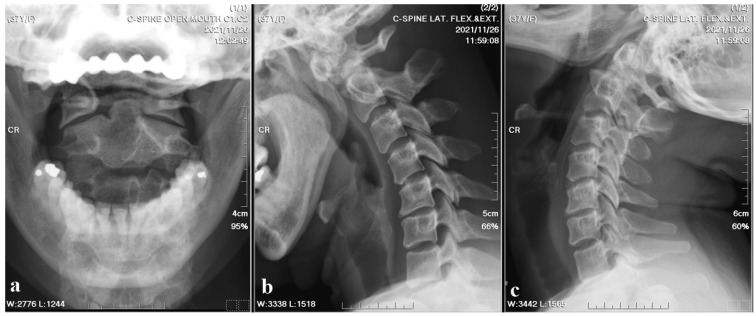
Cervical spine X-ray images. (**a**) Open mouth view revealed the asymmetric widening of the distance between the right C1 arch and the dens with suspected atlantoaxial subluxation. (**b**,**c**) Flexion and extension also showed suspected atlantoaxial subluxation.

**Figure 2:**
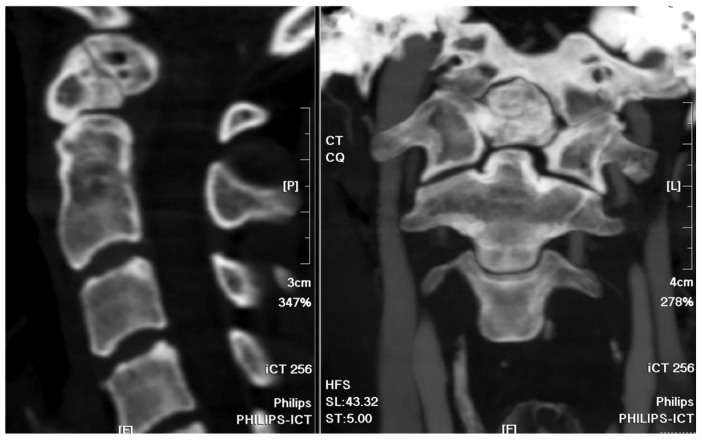
Cervical spine CT bone window images. The sagittal view and coronal view revealed the non-fused C2 bony elements of the odontoid process.

**Figure 3:**
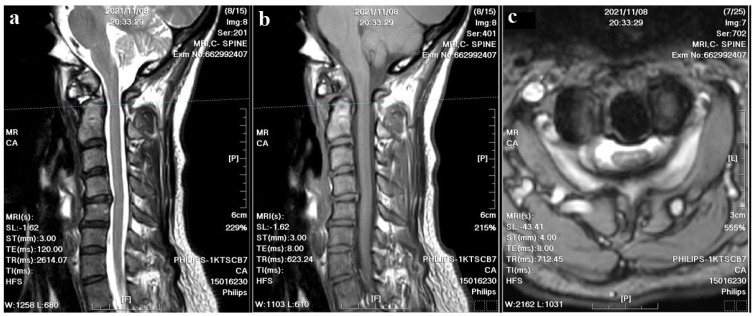
Cervical spine MRI images. (**a**) The T2–weighted sagittal image revealed C1-C2 subluxation and slight spinal cord atrophy; cord edema and inflammatory pannus were also noted at the C1-C2 level. (**b**) The T1–weighted sagittal image showed evident ventral compression at the C1-C2 level. (**c**) The T2-weighted axial image showed a compressed spinal canal at the C1-C2 level.

A 37-year-old female with no prior medical history or spinal surgeries presented to our neurosurgery outpatient department five months after a motorcycle traffic accident. Initially diagnosed with a C2 fracture at a local emergency department, she received only conservative treatment and wore a neck collar. Subsequent to the accident, she experienced persistent neck pain radiating to both shoulders and upper back, right-hand numbness with associated weakness, headaches, neck stiffness, and an unsteady gait. An initial cervical spine X-ray revealed the asymmetric widening of the distance between the right C1 arch and the dens with suspected atlantoaxial subluxation (Figure 1). Computed tomography revealed non-fused bony elements (Figure 2). Further cervical spine magnetic resonance imaging (MRI) (Figure 3) highlighted os odontoideum with C1-C2 subluxation and slight spinal cord atrophy at the C1-C2 level due to instability. There was also evidence of ventral compression at the C1-C2 level from chronic inflammatory pannus and granulation tissue.

For the atlantoaxial subluxation, the patient was prepared for the AR-assisted navigational instrumentation surgery. Preoperative cervical spinal MRI and computed tomography angiography (CTA) were performed to rule out angioarchitecture variation. Post-surgery, the patient exhibited a notable alleviation of her symptoms. Postoperative recovery was uneventful and without neurological deficit, culminating in her discharge on the fourth postoperative day.

## 3. Surgical Technique

**Figure 4:**
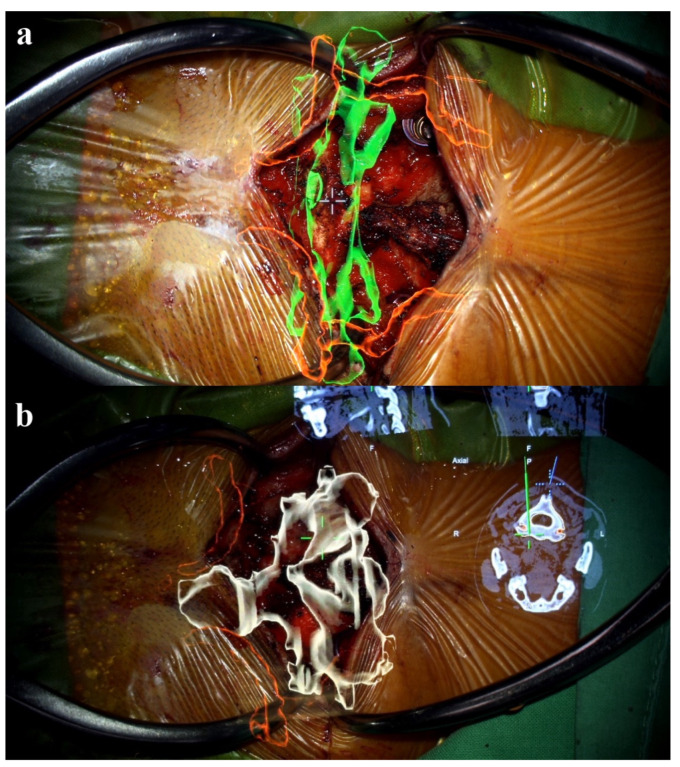
AR projection under the microscope. (**a**) The AR projection showed a spatial relationship between VA (red color-coded) and C1 bony structures (green color-coded). (**b**) Projection of C2 osseus structure (white color-coded) and VA (red color-coded) with real time navigation information.

**Figure 5:**
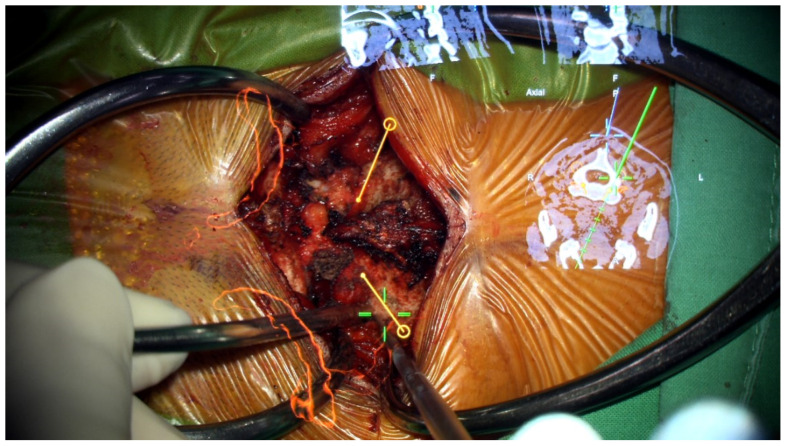
AR-assisted navigation under the microscope. The entry point and pre-set trajectory for C2 instrumentation were projected onto the surgical field.

**Figure 6:**
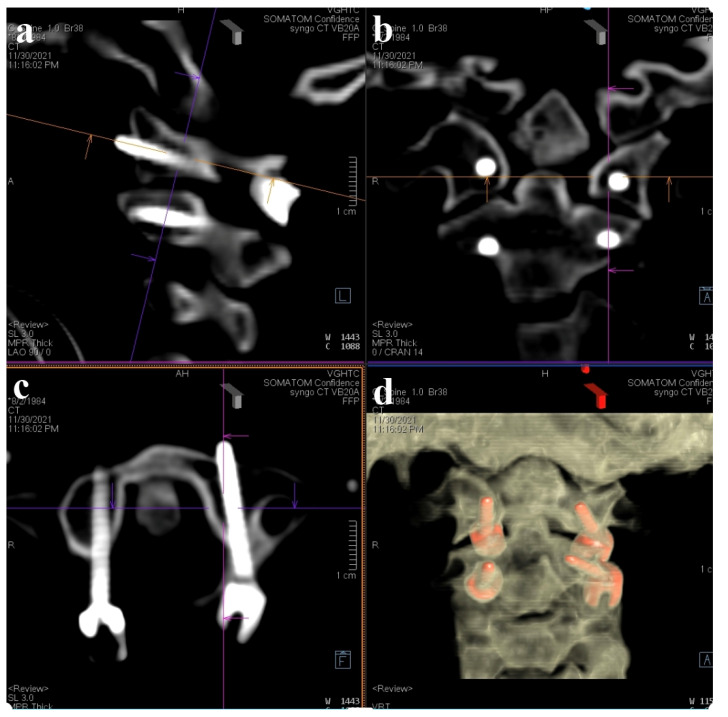
Intraoperative CT for screw validation. (**a**) Sagittal view; (**b**) coronal view; (**c**) axial view at C1 level; (**d**) reconstruction of 3D image.

Preoperatively acquired cervical spinal MRI and CTA data were incorporated into the BrainLab Curve stereotactic neuro-navigation system (BrainLab AG, Munich, Germany). Prior to the procedure, we delineated the bilateral VAs and bony anatomy in distinct colors on a 3D-reconstruction derived from the preoperative CTA. At the surgery’s onset, the patient was secured in a prone position using a skull clamp. A navigation recognition reference cluster was affixed to the head frame for system registration. Subsequent to this, another stereotactic reference was attached to the KINEVO 900 microscope (Carl Zeiss AG, Oberkochen, Germany). An initial intraoperative CT scan was executed, and its imagery was coalesced with the pre-configured navigation images to counteract potential inaccuracies stemming from divergent scan positioning. For the C1-C2 fixation, we determined screw entry points and trajectories utilizing the navigation system, adopting a posterior midline technique. Initially, the lateral facet of the C1 posterior arch was unveiled to discern bilateral–lateral masses. Subsequent exposure included the spinous process, laminae, and the medial segments of C2 lateral masses. Utilizing the AR modality, the bilateral VAs, distinctly marked in red, were distinctly observed under the microscope. Concurrently, projections of the supplementary osseous structure were cast onto the operative field (Figure 4). Subsequently, entry points and trajectories for the C2 pedicular screws were orchestrated using real-time navigation in sync with the microscope-integrated AR system (Figure 5). Upon appropriate magnification and zooming out, the surgeon could clearly visualize the anatomical structures, avoiding any obstruction caused by surgical tools. Validation using AR navigation and intraoperative fluoroscopy confirmed the correct positioning. We proceeded by creating a pilot hole with a 2 mm high-speed burr. Following the pilot hole, we used a tool equipped with a navigator to ensure adequate bone purchase and a safe trajectory for further tapering. Finally, we placed poly-axial C2 pedicular screws (3.5 × 28 mm) along the pre-established trajectory, ensuring no breaches or vascular injuries. A corresponding navigation strategy was employed for the bilateral C1 lateral mass screws (3.5 × 30 mm). Post screw placement, an intraoperative CT scan affirmed the screw alignments (Figure 6). Succeeding surgical phases, encompassing the C1 laminectomy and rod application adhered to conventional protocols.

## 4. Discussion

Real-time image-guided navigation systems have greatly reduced the risks of VA, spinal cord, and nerve root injuries during posterior C1-C2 fixation surgeries [7]. The literature reveals varying incidence rates of VA injury during cervical spine surgeries, attributed to different surgical techniques. For instance, the incidence of VA injury when employing C1-2 trans-articular screws is reported to range from 1.2% to 13.1% per screw [8]. The risk associated with the placement of C1 lateral mass screws to C2 pedicle screws falls between 2.1% and 5% [9,10,11]. In contrast, the incidence of VA injury is notably low with the use of subaxial lateral mass screws. These data underline the diverse risks linked to different cervical spine stabilization techniques and emphasize the need for technique-specific precautions to minimize vascular complications. Notably, the adoption of intraoperative navigation systems has led to a significant reduction in the occurrence of VA injuries [12,13,14].

However, a significant challenge arises due to the potential discrepancy between the anatomical positioning of a patient during surgery (prone) and during preoperative CT scans (supine). This inconsistency can lead to navigation inaccuracies, increasing the potential for intraoperative complications such as screw malposition and injury to critical structures like the vertebral artery or spinal cord. Addressing this issue is crucial for enhancing the safety and effectiveness of surgical procedures [6,14]. To mitigate these risks and enhance navigation precision, we integrated preoperative CTA with intraoperative 3D CT images. This integration allows for a more accurate representation of the patient’s anatomy as it appears during surgery. By processing these images within the navigation software, we were able to verify the anatomical alignment meticulously, ensuring that the preoperative planning accurately reflects the intraoperative reality. This approach allowed us to focus specifically on the relevant cervical spinal segments, providing a detailed and precise guide for the surgical procedure. The combination of preoperative CTA and intraoperative 3D CT imaging thus represents a significant advancement in surgical navigation. It not only improves the accuracy of anatomical localization but also enhances the overall safety and outcomes of cervical spine surgeries. This method demonstrates the importance of integrating advanced imaging techniques to address the inherent challenges posed by patient positioning discrepancies.

With the advancement of optical and electronic technologies, the microscope-based navigation system has shown promising results in spinal surgeries [15]. In a recent study proposed by Lin et al. [16], the precision of C1-C2 screw instrumentation was demonstrated to be feasible in four patients with atlantoaxial instability. Utilizing AR holography projected onto the surgical field, the C1-C2 bony anatomy, as well as the VAs, were vividly visualized under the microscope. This allowed for real-time insight into the spatial relationship between the screw entry point and the path of the VA, bolstering surgeon confidence during screw placement. Moreover, compared to AR-assisted intracranial microsurgery employing a microscope, AR navigation in spinal surgery is less susceptible to problems of intraoperative navigation deviation arising from structural contraction post-cerebral spinal fluid drainage. This advantage not only enhances accuracy but also reduces the need for frequent intraoperative CT scans and subsequent image processing procedures.

In contrast to the microscope-based AR system, traditional optical navigation encounters technical constraints due to line-of-sight challenges. These challenges often arise when optical obstructions prevent the surgical tool’s tracing marker from being captured by the external camera, leading to disruptions in the navigation process [17]. A potential solution to this limitation is the automated calibration between the reference array anchored to the microscope and another reference affixed to the skull clamp, which is positioned away from the surgical field. This approach not only enhances intraoperative precision but also mitigates issues associated with infrared sensor blockage.

Additionally, by utilizing the microscope-based AR system, surgeons can streamline their focus without the need to glance at a separate monitor. With this integrated imaging system, all navigation data are directly relayed to the microscope, merging seamlessly with the anatomical views of the surgical area. This integration proves beneficial by minimizing potential distractions for the surgeon. Nonetheless, surgeons must remain vigilant to ensure that the AR projections align accurately with the actual anatomy.

While this innovative approach offers many advantages, there exist inherent limitations associated with the integration of microscope-based AR navigation in surgical procedures. One of the most significant challenges is the system’s reliance on a surgeon’s adeptness and prior exposure to AR technologies. This dependence means that surgeons must possess a high level of proficiency and familiarity with AR systems to fully leverage their potential. Consequently, the initiation of comprehensive training modules tailored to surgeons, assisting nurses, and radiological technicians becomes essential. These training programs, however, necessitate substantial time and fiscal resources, posing a barrier to widespread adoption. Additionally, the considerable capital expenditure required for the initial setup of microscope-based AR navigation systems is a critical financial constraint. Hospitals and medical institutions must invest heavily in purchasing and installing this advanced technology. Moreover, the ongoing maintenance costs associated with keeping the system operational and up-to-date further exacerbate the financial burden. These costs include regular software updates, hardware maintenance, and potential system upgrades to ensure optimal performance and safety.

Despite these challenges, the integration of AR navigation holds promise for enhancing surgical precision and outcomes. Addressing financial and training-related barriers is crucial for the broader adoption of this technology. Continued investment in education and infrastructure, alongside evidence of improved surgical outcomes, may help justify the costs and facilitate the more widespread use of AR navigation in surgical procedures.

## 5. Conclusions

In this case study, we detail an instance wherein a patient underwent C1-C2 instrumentation surgery utilizing a microscope-based AR navigation system. This patient exhibited a notable alleviation of her symptoms. Postoperative recovery was uneventful and without neurological deficit, culminating in her discharge on the fourth postoperative day. As advancements in AR technology continue to emerge, it is anticipated that such cutting-edge techniques will gain broader traction in both surgical practices and medical education. However, to firmly establish the precision and efficacy of this procedure, more expansive studies are requisite.

## Data Availability

Data are available upon reasonable request. The datasets used during the current study are available from the Taichung Veterans General Hospital; however, restrictions apply regarding the availability of these data, as they are not publicly available. However, the data are available from the corresponding author upon reasonable request and with permission from the Taichung Veterans General Hospital.

## Abbreviations

Augmented reality (AR); cone beam computed tomography (CBCT); computed tomography angiography (CTA); image-guided systems (IGSs); magnetic resonance imaging (MRI); vertebral artery (VA).
